# The Medical Library of the University of Bristol

**Published:** 1958-01

**Authors:** A. E. S. Roberts


### THE MEDICAL LIBRARY OF THE UNIVERSITY OF BRISTOL

BY

A. E. S. ROBERTS

The Medical Library of the University of Bristol is to-day a concentration of all the medical libraries that have been formed in Bristol during the last two centuries. Several of these old libraries were personal collections built up by book lovers and experts in their professional field, and contained many rare and first-class works. This explains how we come to possess such a magnificent collection of the classics of medicine and of early printed books. This combined collection has been in existence since 1893 when the library of the Bristol Medico-Chirurgical Society first became part of the Medical School Library; and to-day the number of volumes in the library has grown to nearly 40,000 and the current periodicals number some 450.

The library of the Bristol Medico-Chirurgical Society was established in 1890 and was formally opened on 5th January, 1891, by the President of the Society, Mr. Samuel Henry Swayne (1820–1900), as a Medical Reading Room and Reference Library in accommodation provided by the Literary and Philosophic Club at 22 Berkeley Square. A start was made with over a thousand volumes (including a large donation from the British Medical Association), and seventy-nine current periodicals, and it quickly grew as the result of gifts from individuals, grants from the Society and review books and exchange journals through the *Bristol Medico-Chirurgical Journal*. At this time the Medical School had no library for its Staff, and did not provide books for either its teachers or its students, so the Society agreed with the University College to form a joint library, and on 1st July, 1893, the Society's library was transferred to the new Medical School buildings.

The library was now housed in a room magnificent in its proportions and decorations, but strangely inadequate for its growth, although it had been specially designed for the purpose. The walls on three sides of the room were occupied for about three-quarters of their height by windows, which, though very beautiful in themselves, considerably lessened the wall-space available for book-shelves and it soon became necessary to erect shelves against the greater part of these windows.

This joint library was known as the Bristol Medical Library and its affairs were controlled by a committee of three members from the Society and three from University College.

The next collections to be added to the Bristol Medical Library were those belonging to the Infirmary and the General Hospital. This incorporation took place in 1894, mainly through the influence and energy of James Greig Smith (1834–1897). The Infirmary Library had been established in 1826 by initial gifts of books by Richard Smith (1772–1843) and Richard Lowe (died 1850). It was not until 1838, however, that a library committee was formed which at its first meeting drew up some library rules. These rules stated that all Infirmary students and pupils could have access to the library and could take out books. The House Surgeon was put in charge of the library and he was authorised to appoint one of the assistant pupils as "Acting Librarian". In the remarks column of the 1855 catalogue of the Infirmary Library (which may still be at the Infirmary) borrowers who refused to return books at the request of the Acting Librarian had written facetiously against the missing books such remarks as "Sold to buy gin", "Deuced odd", "Popped at Chilcott's", "humbug". There is a printed catalogue in the Medical Library of the contents of the Infirmary Library which shows what a magnificent library it was, being particularly rich in eighteenth-century books.

Soon a further collection of books was presented—by the City authorities. This particular library had had a rather chequered history. It was originally called the Bristol Medical Library when it was founded as a subscription library by Edward Kentish and others in 1832. It was housed in a building in Orchard Street, leased from the Corporation at a yearly rental of two pounds. The library remained there until 1856 when it was transferred to the custody of the Bristol Library, another and older subscription library founded in 1772, which had just moved from King Street to the west wing of the Bishop's College which then stood on ground afterwards occupied by the Art Gallery. The Bishop's College ceased to exist in 1861 and the property passed to the ownership of the Bristol Rifles Headquarters Company, but the libraries were able to remain there till 1871, when they were moved to the Museum and Library in Queen's Road. The Museum and Library did not receive the public support that was expected and in 1893, through the generosity of the shareholders and the liberality of Sir Charles Wathen (1832–1893), a former Mayor, who undertook the payment of all outstanding liabilities, it passed to the ownership of the City. Soon afterwards the City authorities handed over to the Medical School the medical portion of the library which, of course, contained many of the books from the old Orchard Street collection.

The latest addition to the Medical Library of the University came from the Royal United Hospital, Bath, in 1950. This was a collection of medical libraries gathered together by Bath medical men, mainly by Caleb Hillier Parry (1755–1822) and John Smith Soden (1780–1863) but it had not been added to for many years. The Parry part has a separate manuscript sheaf catalogue and these books are kept together as the "Parry Collection", while the others have been absorbed into the general arrangement of the Medical Library. Though some of the volumes are in poor condition, the collection is rich in rarities and old classics.

Of all these libraries, only the library of the Bristol Medico-Chirurgical Society is still alive. The *Bristol Medico-Chirurgical Journal* (now renamed the *Medical Journal of the South-West*) plays a large part in the growth of the library, for all books reviewed in the *Journal* and all periodicals received in exchange are placed in the library. In 1948 the *Journal* ceased to publish book reviews because the price of printing was too high, and so review books are no longer received in the library, but the Society, to cover this loss of review books, makes an annual grant to the library. Exchange periodicals still come to the library; these include some out-of-the-way items, and while the library would not purchase all of them if they were not received free, some of them nevertheless form a valued part of the library in these days of extensive library co-operation, because they are possessed by very few of the other libraries in the country.

The library was moved again in 1912 because the University required the library room for a Council Chamber. Accommodation was provided in the east wing of the Blind Asylum, but here the library's stay was short, for in 1915 the building had to be pulled down to make way for a new University library and administrative block, and it was necessary once more to find other quarters for the Medical Library. This was arranged in the old Drill Hall, the entrance to which was from University Road. It was not thought the Medical Library would remain here for long but in fact the library part of the new building was not ready for occupancy until 1923 in which year the medical books were transferred to their present home. An extension of the library wing of this block was completed by 1940, although it was not used for library purposes till after World War II in 1945. The Medical Library's share of this extension is used as a stackroom.

L. M. Griffiths (1845–1924), the first Honorary Librarian of the Bristol Medico-Chirurgical Society, was succeeded in 1902 by C. King Rudge (1846–1926), and after him there followed W. Alexander Smith, George Parker and A. Rendle Short. Between 1938 and 1950 no Honorary Librarian was appointed by the Society but the Professors of Medicine, Surgery and Obstetrics were asked to act in that capacity. The present Honorary Librarian is Dr. H. J. Orr-Ewing.

From 1912 to 1922 the Medical Library staff, although appointed by the University, was under the orders of the Honorary Medical Librarian, and the library was managed by a committee of ten members, five appointed by the University and five by the Society. On 21st June, 1922, the Society, at a special meeting, unanimously passed a resolution to make an absolute gift of its library to the University, subject to conditions which would ensure members of the Society, present and future, enjoying full privileges of the library. A new agreement was completed on 9th December, 1925, between the Society and the University which provided, among other things, "That the Medical Library shall be under the ultimate control of the Librarian of the University who shall be responsible to the Library Committee of the University. That the general management of the University Medical Library shall be vested in the University Council who may delegate to the Library Committee power to make and alter Regulations for using the same. There shall be a special Sub-Committee of the Library Committee to be known as the "Medical Library Sub-Committee", which shall report directly and be responsible to the Library Committee. That the constitution of the Medical Library Sub-Committee shall be as follows:—"The Chairman of the Library Committee, the Librarian, the Dean and one other representative of the Faculty of Medicine, two representatives of the Society and the Honorary Medical Librarian who shall be appointed by the Society". "The office of the Honorary Medical Librarian shall carry no administrative authority, but shall be of an advisory character only".

To-day in 1957, we are contemplating a future move of the Medical Library to the new Medical School, even though the building has not yet progressed beyond the architect's drawing board.

### REFERENCES

Smith, G. Munro: *A history of the Bristol Royal Infirmary*, Arrowsmith, Bristol, 1917.
Various articles in the *Bristol Medico-Chirurgical Journal.*

